# 

**Published:** 1850-05

**Authors:** 


☞ Several editorial articles are excluded from this No. by the *title page and index.* We have received the Transactions of the State Med. Society; report of committee on Med. Colleges to the Senate on Epidemic Cholera, and several other publications, which will receive notice in our next No.

Communications from Drs. George Hadley, F. H. Hamilton, C. Colgrove, and P. H. Strong, have been received and will appear in our next No.

☞ See cover for correction of errors in the article by Prof. Hamilton in the present No.

